# Supplementary material for: Rapid turnover of life-cycle-related genes in the brown algae
Source: Genome Biol. 2019 Feb 14;20:35. doi: 10.1186/s13059-019-1630-6 (PMC6374913; doi:10.1186/s13059-019-1630-6)
Supplement: Supplementary file 3 — Protein alignments, corresponding Gblocks html file and CDS sequences for the lineage conserved gametophyte-biased genes. (ZIP 2109 kb) [file 13059_2019_1630_MOESM3_ESM.zip › Con_GA_alignments/Ec-10_001260.aa_ali.fasta-gb.htm]

Ec-10\_001260.aa.fasta.trans.aa\_ali.fasta


## Gblocks 0.91b Results

Processed file: **Ec-10\_001260.aa.fasta.trans.aa\_ali.fasta**  
Number of sequences: **2**  
Alignment assumed to be: **Protein**  
New number of positions: **596** (selected positions are underlined in blue)

```
                         10        20        30        40        50        60
                 =========+=========+=========+=========+=========+=========+
Ec-10_001260     MSRSDIVEEQKYAGENEASGGLLEESAPGAPTRHGGRENIKVLVRIRPLLPNEAGPVAVV
MSTRG.15671.1    MSR---VED--AAGTPEVSRSLDSDSSDDS-IEISGRESIKVLVRIRPLLAHEIGPAVVQ
                             ##############         #########################


                         70        80        90       100       110       120
                 =========+=========+=========+=========+=========+=========+
Ec-10_001260     IGEGNTIRVKGATAQRQLQCRYDAVIGSEVSQEGVFSHVRECTSAVLEGENSSIFAYGQT
MSTRG.15671.1    EGEENMIRVTG--ALQQLHCRYDAVIGADVSQEGMFSHVRECTSAVLRGENSTIFAYGQT
                 ###########  ###############################################


                        130       140       150       160       170       180
                 =========+=========+=========+=========+=========+=========+
Ec-10_001260     GSGKTYTMFGPDLDANGGTAPGYGKGIIPLAVTDLFQGLAGLRQEMGGAGESSVWCSGVQ
MSTRG.15671.1    GSGKTYTMFGSDSDGNGGAPSAHAKGVIPLAVADLFERLSDTGRQED-HDNASVWMSLVQ
                 #######################################             ########


                        190       200       210       220       230       240
                 =========+=========+=========+=========+=========+=========+
Ec-10_001260     IYNEQLFDMLRDPQRIHPLAIHEQAEAGIYVQGLSEYAVRSARECLQLLRVGREHRAIRE
MSTRG.15671.1    LYNEQLYDMLRDPQRVHPLAIHEEEGAGIYVQGLSEYAVNSAGECLQLLRVGREHRAIRE
                 ############################################################


                        250       260       270       280       290       300
                 =========+=========+=========+=========+=========+=========+
Ec-10_001260     THMNQASSRSHSIFQIVVEQKRRSEQDGERILRSKFNLVDLAGSEKWDVKQDMAEVRVSE
MSTRG.15671.1    THMNQASSRSHSIFQIVVEQKRQSEEGGECVLRSKFNLVDLAGSEKWDVKQEMEEVRVSE
                 ############################################################


                        310       320       330       340       350       360
                 =========+=========+=========+=========+=========+=========+
Ec-10_001260     MTNINVSLYTLGRVIAALSTPNSHGEGGARKGHVPYRDSKLTRLLQDSLGGNTRTRIIAT
MSTRG.15671.1    MTNINVSLYTLGRVIATLCR----SEGGSLKGHIPYRDSKLTRLLQDSLGGNTRTRIIAT
                 ##################       ###################################


                        370       380       390       400       410       420
                 =========+=========+=========+=========+=========+=========+
Ec-10_001260     LSPASQSVDETISTLRFADRAKQVMAFVRVNERRPVDHALVHRLQAEVNHLRGLVQDTAE
MSTRG.15671.1    LSPAKQCLDESISTLRFADRAKQVMTFVRVNEQRPVDHALVHRLQAEVKHLRRLLRETAG
                 ############################################################


                        430       440       450       460       470       480
                 =========+=========+=========+=========+=========+=========+
Ec-10_001260     ARSLREEAPDGPTRPPALTALAEAQRLGYQETIDQLRRENTALRENQGPKAVDKSDNKPG
MSTRG.15671.1    ERGLQNETPTTTLRPAALTALAEAQKLGYQETIKQLRRENTALREKLHRNVVVNGPGNQE
                 ############################################################


                        490       500       510       520       530       540
                 =========+=========+=========+=========+=========+=========+
Ec-10_001260     QMESDVATLQAANRALSTAIEHIVLDIRRFFRFDIEEEDLRADLAKLLTQLSGSIPGGAR
MSTRG.15671.1    QTGLDVDALQAANRTLKGAIEHIVVNVRRFFRFEVEEEDLRGDIADVLTGLNGSVLDATT
                 ############################################################


                        550       560       570       580       590       600
                 =========+=========+=========+=========+=========+=========+
Ec-10_001260     WCNSLNVSTSNSCSALAAAQQTIRQGIDAQEHSNSHEKSTPGEAQSETRGGLPAASQLWK
MSTRG.15671.1    WCEAPKCARRNSSRVLAGAS-------AAQRGRNLHDSDFLENRKDGGRG-K--------
                 ###################                                         


                        610       620       630       640       650       660
                 =========+=========+=========+=========+=========+=========+
Ec-10_001260     GGVAAGGQLHQEKSQSEHVALPPV---TPKKDHTSEKLAYRLRGKHADEMVVVMKKEITE
MSTRG.15671.1    ------PYQQEEISLSERITLPPVSGVAPEKEARPGKLAYRLRGKHADEVVVVMRREATE
                            #############    ################################


                        670       680       690       700       710       720
                 =========+=========+=========+=========+=========+=========+
Ec-10_001260     KDEELQLRKELRLAKERMKKNIQLQAWLLQKEQREIEMLKSESEKRDELDRDRRRKDEEF
MSTRG.15671.1    EDEERQLRKDLKLAKERMKKNIQLQEWLLQKERMEK------------------------
                 ###################################                         


                        730       740
                 =========+=========+======
Ec-10_001260     LRRAAKQKAKLKRYCKGRLLRILSHA
MSTRG.15671.1    --------------------------
```

```
Parameters used
Minimum Number Of Sequences For A Conserved Position: 2
Minimum Number Of Sequences For A Flanking Position: 2
Maximum Number Of Contiguous Nonconserved Positions: 8
Minimum Length Of A Block: 10
Allowed Gap Positions: None
Use Similarity Matrices: Yes
```

```
Flank positions of the 7 selected block(s)
Flanks: [13  26]  [36  71]  [74  159]  [173  318]  [326  559]  [612  624]  [629  695]  

New number of positions in Ec-10_001260.aa.fasta.trans.aa_ali.fasta-gb:  596  (79% of the original 746 positions)
```
